# Role of exosomal competitive endogenous RNA (ceRNA) in diagnosis and treatment of malignant tumors

**DOI:** 10.1080/21655979.2022.2073130

**Published:** 2022-05-16

**Authors:** Mingwen Mao, Jingyu Zhang, Yizhen Xiang, Mengdan Gong, Yongqin Deng, Dong Ye

**Affiliations:** a Department of Otorhinolaryngology-Head and Neck Surgery, Lihuili Hospital of Ningbo University, Ningbo, China; b Department of Otorhinolaryngology, NingboNo.6 Hospital Ningbo, China; c Department of Otorhinolaryngology-Head and Neck Surgery, Shanghai Jiao Tong University, Shanghai, China

**Keywords:** ceRNA, malignant tumor, exosomal, diagnosis, treatment

## Abstract

Malignant tumors are a threat to human health, thus it is critical to better understand the mechanism of tumor occurrence and development and to find key therapeutic targets. Competitive endogenous RNA (ceRNA) is a type of RNA molecule that includes mRNA of coding-protein, pseudogenes, long non-coding RNA (lncRNA), and circular RNA (circRNA) etc. It is created through a competitive combination of common small RNA (miRNA) and has an inhibitory effect on mRNA translation. ceRNA regulate the post transcriptional expression of genes by competitively binding to common microRNAs (miRNAs).Studies have shown that cernas are involved in tumor cell proliferation, invasion and migration, drug resistance, angiogenesis, as well as tumor immunity, and so on, affecting the progression of tumor development. This article reviews the reported roles of exosomal ceRNA in the diagnosis and treatment of malignant tumors and the mechanisms underlying these.

## Graphical abstract

**Figure:**
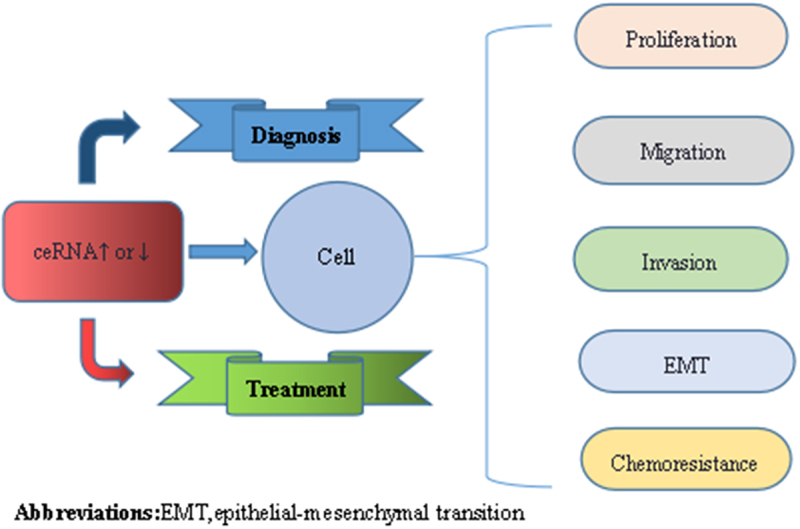

## Highlights

- Exosomal ceRNAs regulate the occurrence and development of malignant tumors.
- Exosomal ceRNAs can be used as a diagnostic marker for malignant tumors.
- Exosomal ceRNAs can be targets for immunotherapy of malignant tumors.

## 1. Introduction

Malignant tumors are one of the leading causes of death worldwide. Cancer morbidity and mortality are rising and cannot be ignored. As a result of the absence of effective early diagnostic markers and detection methods, many patients are diagnosed late, when the 5-year survival rate is considerably lower. It was previously believed that a malignant tumor is formed when original cancer genes are activated, leading to abnormal tumor cell proliferation. However, a recent study found that ceRNA is expressed in a variety of malignant tumors, impacting tumor occurrence, development, and sensitivity to targeted drugs.

A exosomes is a membranous sac secreted by intracellular multivesicular bodies and released into the extracellular matrix after cell membrane fusion. The surface consists of polysaccharides, protein receptors, and other lipids with a double structure, and they contain many biologically active substances [1]. The exosomes contains protein, mRNA of coding-protein, and miRNA secretion signal molecules that reflect the physiological and functional state of the secretory cells. Recent studies show that tumor cells secrete ceRNA into saliva, cerebrospinal fluid, blood, and other body fluids [2].

Exosomal ceRNA contains mRNA of coding-protein, pseudogenes, long noncoding RNA, and circular RNA etc. Several studies have confirmed that dysregulation of cernas is closely related to tumorigenesis, development and chemotherapeutic drug resistance in breast cancer, gastric cancer, lymphoma and other cancers.The research of ceRNA deeper, it can promote the development of clinical diagnostic technology of tumors and immunotherapy of tumors.

## 2. Exosomal ceRNA composition and biological functions

### 2.1 The exosomal ceRNA composition

**Figure 1.**
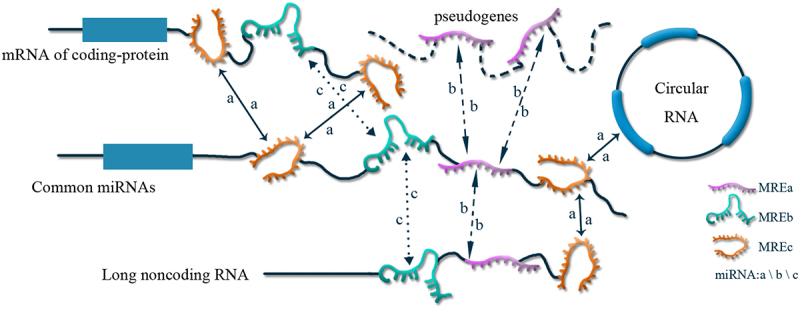
The regulatory mechanism of exosomal ceRNA.

Recently, a new hypothesis about how long non-coding RNAs and mRNAs ‘communicate,’ was proposed and named the ceRNA regulation hypothesis because of its role in cell regulation. The discovery of how ceRNA regulates cells shows that mRNAs do not merely encode proteins but are also involved in regulating gene expression. In addition to microRNA (miRNA) binding to target genes and affecting RNA stability and translation processes at the post-transcriptional level, RNAs can also, in turn, affect miRNA levels. Various types of RNA transcripts use miRNA response elements (MREs) to achieve mutual regulation by competing for binding to common miRNAs and influencing expression of free miRNAs. Meanwhile, the more miRNA species are shared between two RNAs, the stronger the competitive endogenous relationship. Based on ceRNAs regulatory mechanisms, large-scale transcriptional regulatory networks based on ceRNAs are established, enriching signaling networks and expanding functional genetic information in the human genome. This provides a new theoretical basis for the in-depth study of tumor pathogenesis[3]. The regulatory mechanisms of exosomal ceRNA are shown in Figure 1.

Like proteins, exosomal ceRNA have three basic levels of RNA structure: primary, secondary, and tertiary, which refers to linear multimers in which ribonucleotides are linked to each other by 3 ′, 5 ′-phosphodiester linkages, and the order in which they are arranged. Common secondary structures include double-stranded, hairpin, bulge, inner loop, and linker structures, which can provide binding sites for a variety of proteins. RNA molecules further distort and fold based on their secondary structure to form tertiary structures [4].

Exosomal ceRNAs include mRNA of coding-protein, miRNAs, pseudogene transcripts, long noncoding RNAs (lncRNA), and circular RNAs (circRNAs) etc [3]. MiRNAs, are a class of non-coding regulatory single-stranded small molecular RNAs with a very short length, approximately 22 NT, that serve as transcripts of coding genes. Pseudogenes are a class of genes that are similar in sequence to functional genes but unable to encode functional proteins because of mutations and loss of promoter sequences. Long non-coding RNAs (lncRNA) are a group of non-protein-coding RNA molecules over 200 NT. Circular RNAs (circRNAs) are novel endogenous noncoding RNAs with a closed-loop structure generated from protein-coding genes through a back splicing process without polar and polyadenylated tails [5].

### 2.2 Biological functions of exosomal ceRNAs

#### 2.2.1 Regulation of tumor cell proliferation

In the tumor cells, the dysregulation of ceRNA will lead to the amplified dysregulation of downstream regulators and target molecules. Finally, this cascaded dysregulation of ceRNA network strongly promotes the malignant progression of malignant tumors [6,7].

Exosomal ceRNAs are key mediators of communication between tumor cells and surrounding cells [8], and malignant tumor cell-derived exosomal ceRNAs play an important role in tumor progression. CeRNAs may regulate cellular communication by transferring miRNAs between different cells. Dysregulated expression of ceRNAs that target different miRNA targets in malignant tumors results in tumor cell proliferation by reducing apoptosis or dedifferentiation and ultimately leads to tumor growth [9–11].

#### 2.2.2 Regulation of tumor cell development, invasion, and metastasis

During tumor metastasis, tumor cells invade other normal organs of the body to form new metastatic foci. The basis of malignant tumorigenesis is epithelial-mesenchymal transition (EMT), which enables epithelial cells generated in specialized sites to detach from epithelial tissues and migrate to other locations. The EMT phenomenon was seen in tumors from various pathological stages. Exosomal ceRNAs can regulate this process by sequestering and regulating miRNAs involved in EMT-related signaling pathways to promote tumor cell metastasis and invasion [4,11,12].

Hypoxia results in the activation of inducible factors that lead to malignant tumor angiogenesis. Tumor cells exhibit greater growth in hypoxic environments, secreting higher ceRNA levels in exosomes, which in turn regulate the tumor microenvironment and promote tumor angiogenesis and metastasis [13].

#### 2.2.3 Modulating tumor chemotherapy sensitivity

Exosomal ceRNAs regulate the sensitivity of malignant tumors to chemotherapeutic drugs by binding target genes and causing direct sponging, or repression, using epigenetic mechanisms. They primarily exhibit secondary or acquired resistance to chemotherapy by inactivating or altering target cells, inhibiting cell death using epigenetics, EMT, and other mechanisms [14]. This alters the sensitivity of tumors to chemotherapy by changing ceRNA expression.

## 3. ExosomalceRNAs in the diagnosis and treatment of malignant tumors

### 3.1 Role of exosomal ceRNAs in the diagnosis of malignant tumors

Changes in ceRNA expression occur in many types of malignant tumor, and these changes can be used to predict clinical diagnosis and tumor prognosis. The following summarizes the role of different ceRNAs as clinical diagnostic markers across systems:

CircRASSF2 expression was significantly elevated in laryngeal squamous cell carcinoma (LSCC) tissues and LSCC plasma exosomes. CircRASSF2 enhanced LSCC cell proliferation, migration, and invasion by regulating mir-302b-3p/insulin-like growth factor 1 receptor (IGF-1 R). CircRASSF2 may thus serve as a clinical molecular diagnostic marker for LSCC [15]. CircFNDC3B was upregulated in papillary thyroid cancer (PTC) tissues and cell lines. Tumor size, lymph node metastasis, tumor invasion, and the stage of advanced tumor node metastasis (TNM) were associated with higher expression of circFNDC3B. CircFNDC3B was also shown to promote cell proliferation, migration, invasion, and reduce apoptosis in vitro. The mechanism by which circFNDC3B exerts its malignant effects was verified by bioinformatics analysis, combined with biological experiments. CircFNDC3B, as a competing endogenous RNA (ceRNA), regulates PTC progression through the mir1178/TLR4 pathway. CircFNDC3B can thus be a target for PTC diagnosis [16].

CircRNA-002178 was found to be upregulated in lung adenocarcinoma cells using functional analysis, and upregulated circRNA-002178 was detected in exosomes from the plasma of lung adenocarcinoma patients. Thus, circRNA-002178 could be used as a potential noninvasive biomarker for early detection of lung adenocarcinoma [17].

lncRNAFAL1 was upregulated in HCC tumor tissues, cells, and serum exosomes from HCC patients. lncRNAFAL1, as an oncogene, promotes cancer cell proliferation and metastasis by competitively binding with mir-1236, which in turn upregulates expression of its target genes, AFP and ZEB1. Circulating exosome-mediated lncRNAFAL1 transfer can increase the proliferation and migration of liver cancer cells. These findings suggest that lncRNAFAL1 plays an oncogenic role in liver cancer and may have implications for the development of novel diagnostic biomarkers in the future [18]. CircNRIP1 was significantly upregulated in human gastric cancer (GC) tissues and promoted the proliferation, migration, and invasion of GC cells, and circNRIP1 expression levels were also significantly associated with tumor size and lymphatic invasion. CircNRIP1 may thus be useful as a gastric cancer diagnostic or prognostic marker [19]. Exosomal circPED8A is highly expressed in pancreatic ductal adenocarcinoma (PDAC) cell tissues, and high levels of exosomal circPED8A are associated with lymphatic invasion, advanced TNM stage, and poor prognosis in PDAC patients. Subsequent studies revealed that tumor-released exosomal circPED8A acts as an ‘oncogene’ by sponging mir-338, upregulating met through the circPED8A/mir-338/MACC1/met axis. Therefore, exosomal circPED8A may be a useful marker for PDAC diagnosis or progression [8]. CircIARS expression was upregulated in both pancreatic cancer tissues and plasma exosomes. CircIARS promotes tumor invasion and metastasis by exosomes entering human umbilical vein endothelial cells (HUVECs), and its expression is positively correlated with liver metastasis, vascular invasion, and TNM stage and negatively correlated with postoperative survival time. CircIARS overexpression significantly reduces expression of miR-122 and ZO-1, upregulates RhoA and RhoA GTP, increases F-actin expression and focal adhesion, enhances endothelial monolayer permeability, and promotes tumor invasion and metastasis. The presence of circIARS in exosomes is a promising biomarker for early diagnosis, metastasis assessment, and PDAC prognosis [8]. Circ-ITCH overexpression could inhibit gastric cancer cell proliferation, migration, and invasion. Mechanistically, circ-ITCH acted as a sponge for mir-199a-5p and regulated the mir-199a-5p/Klotho axis, thereby affecting the EMT process in gastric cancer. Findings suggest that circ-ITCH plays a key role in the progression of gastric cancer and serves as a potential biomarker [20]. Colorectal cancer (CRC) – associated long non-coding rnalinc02418 is an oncogenic lncRNA that promotes CRC cell proliferation and inhibits cell apoptosis through the mir-1273 g-3p-melk axis. Serum exosomal linc02418 may serve as a novel diagnostic biomarker for gastric cancer [21].

CircRNA051239 expression was significantly enhanced in ovarian cancer tissues and plasma exosomes. Studies have elucidated the mechanism by which exosomal circular rna051239 promotes tumor progression of EOC, and circRNA051239 provides a tumor marker for epithelial ovarian cancer (EOC) [22].

Sun et al. [23] explored the tumor markers associated with bladder cancer by constructing ceRNA regulatory network, and successfully screened out several characteristic target molecules, and two lncRNA, linc01198 and pptrd-as1, one miRNA, has-mir-216a, and three mRNAs, sema3d, EphA5, and dclk1, were included in this regulatory network. These RNAs could be considered characteristic target molecules for bladder cancer. CircRNA_103809 is highly expressed in bladder cancer (BC) tissues and cell lines, and is associated with poor prognosis in BC patients. CircRNA_ 103,809 knockdown decreases BC cell growth and metastasis. Thus, circRNAs_103809 could serve as a marker for bladder cancer (BC) diagnosis [8]. Hypoxia-induced lncHILAR, a ceRNA for mir-613/206/1-1-3p, increased invasion and metastasis of renal cell carcinoma (RCC) cells, which resulted in upregulation of Jagged-1 and c-x-c motif chemokine receptor 4 (CXCR4). Activation of the Jagged-1/Notch/CXCR4 axis can induce RCC cancer metastasis. Hypoxia-induced lncHILAR promotes RCC cell invasion and metastasis via ceRNA of mir-613/206/1-1/3P/Jagged-1/Notch/CXCR4 axis. Thus, lncHILAR may serve as a potential biomarker for renal cell carcinoma [24].

### 3.2 Role of exosomal ceRNAs in the treatment of malignant tumors

In today’s era of personalized tumor therapy, harnessing the immune system to fight cancer is an increasingly effective option that can result in dramatic and durable responses across multiple cancers. In recent years, tumor immunotherapy has achieved remarkable success in clinical practice. Exosomal ceRNAs play both suppressive and promoting roles in the occurrence and development of malignant tumors, so they could theoretically function as clinical therapeutics by altering expression of the corresponding ceRNAs. The therapeutic role of exosomal ceRNAs in various systems is summarized below.

The ceRNA network also plays a role in the regulation of immune responses in papillary thyroid carcinoma (PTC) and several miRNAs, such as mir-355-5p, mir-328-3p, mir-183-3p, and mir-29b-2-5p, have been found to suppress activity in PTC. Some of these miRNAs, like mir-7-5p, also have specific immune regulatory profiles. Its target mRNAs acted on recombinant human cardiotrophin-like factor 1 (CLCF1) and insulin receptor substrate 2 (IRS2), and these mRNAs also appeared in the mir-7-5p-mediated ptcceRNA network, suggesting that the PTC immune response was regulated by the ceRNA network. The key RNAs in these ceRNA networks may impact immunotherapeutic outcomes and serve as potential biomarkers [22]. CircFNDC3B was upregulated in the serum exomes of patients with papillary thyroid cancer (PTC). CircFNDC3B regulates PTC progression through the mir1178/TLR4 pathway. CircFNDC3B may therefore be a promising therapeutic target for the treatment of PTC patients [16]. CircRASSF2 also acts as an oncogenic factor in laryngeal squamous cell carcinoma (LSCC), promoting tumorigenesis and progression by sponging mir-302b-3p to regulate IGF1R expression. Thus, the circRASSF2/mir-302b-3p/IGF-1 R axis may provide a novel therapeutic target for LSCC in the future [15]. Exosomal lncRNA HEIH was significantly upregulated in the SCC4/S and DDP resistant tongue squamous cell carcinoma (TSCC) cell line. Downregulation of lncRNA HEIH could inhibit DDP resistance and cell proliferation by promoting cell apoptosis. lncRNA HEIH acts as a competing endogenous RNA (ceRNA) for mir-3619-5p to upregulate HDGF expression. exosomal lncRNA HEIH promotes cell proliferation and drug resistance and inhibits cell apoptosis by sponging mir-3169-5p and upregulating HDGF. It is proposed that exosomal lncRNA HEIH may be a promising therapeutic target for tongue squamous cell carcinoma [25].

Acquired drug resistance is a concern for effective clinical treatment of glioblastoma multiforme (GBM). One study employed QRT PCR and fish to detect LncSBF2-AS1 levels in TMZ-resistant or TMZ-sensitive GBM tissues and cells. LncSBF2-AS1 was upregulated in TMZ-resistant GBM cells and tissues. This miRNA regulated XRCC4 expression by post-transcriptionally regulating mir-151a-3p, and while LncSBF2-AS1 overexpression promoted TMZ resistance, LncSBF2-AS1 inhibition sensitized resistant GBM cells to TMZ. GBM cells remodel the tumor microenvironment and promote tumor chemoresistance by secreting LncSBF2-AS1-enriched exosomes. LncSBF2-AS1 may serve as a therapeutic target for glioblastoma treatment [26].

In the lung adenocarcinoma B cell immune infiltration related ceRNA network [22], three key mRNAs (PBK, KIF23 and SLC7A11) were shown to be significantly associated with survival. They all play a role in B lymphocyte activation, and PBK promotes cell cycle progression, inhibits apoptosis, and promotes promyelocyte proliferation [27]. The lncRNA in the network also had some associations with the survival of lung adenocarcinoma patients, of which AP002478.1, C20orf197, HOTTIP, LINC00337 and MUC2 interacted with several miRNAs in the ceRNA network, thus indirectly acting on target mRNAs. This suggests that key RNAs in the ceRNA network may have some impact on immunotherapeutic outcomes and may serve as potential biomarkers for treatment. CircRNA-002178 was significantly upregulated in lung adenocarcinoma (LUAD) tissues, cancer cells and serum exosomes. Studies indicate that circRNA-002178 can promote lung adenocarcinoma development by sponging miR-34 and regulating mir-28-5p, thereby increasing PDL1 expression. CircRNA-002178 downregulation can be used as a target for immunotherapy[17]. lncRNA UCA1 is increased in gefitinib resistant cells and patient tissues, and extracellular lncRNA UCA1 promotes gefitinib resistance of Non-small Cell Lung Cancer(NSCLC) cells by packaging them into exons. Specifically, lncRNA UCA1 induces gefitinib resistance in non-small cell lung cancer by sponging miR-143. Bioinformatics analysis showed that the lncRNA UCA1/miR-143/fosl2 axis was present in gefitinib resistance of NSCLC. The direct interaction of lncRNA UCA1, miR-143, and fosl2 was verified using a dual luciferase reporter system and rip assay. Thus, targeting the lncRNA UCA1/miR-143 pathway may provide a novel therapeutic strategy to combat resistance to TKIs [28].

In terms of hepatocellular carcinoma (HCC), scholars have previously established lncRNA-circRNA-mRNA functional network of ceRNA network, in which mRNAs are associated with protein phosphorylation, signal transduction, regulation of biological processes such as cell proliferation, transcriptional regulatory activity, double stranded DNA binding and other molecular functions. LncRNA and circRNAs may all have cis- and/or trans-regulatory roles. These specifically expressed RNAs, especially those validated from HCC patient samples, could serve as potential therapeutic targets [29]. CircNRIP1 was significantly upregulated in human gastric cancer tissues and found to sponge mir-149-5p, thereby promoting the proliferation, migration, and invasion of GC cells. Inhibition of circNRIP1 was shown to block the malignant development of GC cells via AKT1/mTOR signaling. Thus, circNRIP1 inhibition will be a promising therapeutic target in GC in the future [19]. lncRNA UCA1 is upregulated in colorectal cancer tissues and functions as an oncogene in colorectal cancer. lncRNA UCA1 can act as a ceRNA to regulate Myo6 expression by competing for miR-143 binding. Upregulation of circulating exosomes in colorectal cancer (CRC) patients can transfer lncRNA UCA1 into CRC cells, promoting cell proliferation and migration. lncRNA UCA1 is an oncogene in CRC that may serve as a candidate target for new treatments of human CRC by inhibiting cebpb, the transcriptional activator of lncRNA UCA1 [30]. LncRNAFAL1 was upregulated in tumor tissue, cells, and serum exosomes from HCC patients. lncRNAFAL1 promotes cancer cell proliferation and metastasis by competitively binding with mir-1236 and upregulating expression of its target genes, AFP and ZEB1. These findings suggest that lncRNAFAL1 plays an oncogenic role in liver cancer and may be of great value for the development of novel therapeutic targets in the future[18]. Knockdown of lncSPRY4-IT1 significantly inhibited gastric cancer (GC) cell proliferation by causing G1 arrest and promoting cell apoptosis, whereas overexpression of lncSPRY4-IT1 promoted cell growth. Bioinformatics analysis predicted that there was a lncSPRY4-IT1/mir-101-3p/AMPK axis during GC progression. A dual luciferase reporter system validated the direct interaction of lncSPRY4-IT1, mir-101-3p, and AMPK. Western blot confirmed that suppression of lncSPRY4-IT1 decreased AMPK expression. Moreover, suppression of lncSPRY4-IT1 inhibited GC occurrence and progression *in vivo*. lncSPRY4-IT1 was also upregulated in the serum exosomes of gastric cancer patients and correlated with cancer metastasis. Thus, lncSPRY4-IT1 may serve as a potential therapeutic target for gastric cancer [31].

CircRNA051239 expression was significantly enhanced in ovarian cancer tissues and plasma exosomes. In addition, circRNA051239 is contained in exosomes released by highly metastatic ovarian cancer SKOV3 cells. IP cells promoted proliferation, stimulated migration, and invasion of low metastatic ovarian cancer SKOV3 cells. Studies show that circRNA051239 affects prss3 expression by sponging mir-509-5p. Recent studies have described the mechanism by which circRNA051239 promotes tumor progression of EOC and provides a potential therapeutic target[22]. Exosomal lncRNAHNF1AAS1 was upregulated in the DDP resistant cell line, HeLa/DDP, and lncRNAHNF1AAS1 silencing inhibited cell proliferation and promoted cell apoptosis in cervical cancer. LncRNAHNF1AAS1 acted as a ceRNA for mir34b to promote tuft1 expression, promoting the proliferation and drug resistance of cervical cancer cells, and inhibiting apoptosis. Downregulating lncRNAHNF1AAS1 may be a new option for cervical cancer treatment [32]. The circHIPK3/mir-582-3p/RNF11 axis enhances chemoresistance in breast cancer cells, and exosomal circHIPK3 improves trastuzumab resistance in trastuzumab-sensitive breast cancer cells. Thus, circHIPK3 may serve as a therapeutic target in breast cancer [33]. A novel HIF-2 target lncRNA, RAB11B-AS1, was recently discovered in breast cancer cells. RAB11B-AS1 enhanced the expression of angiogenic factors like VEGFA and ANGPTL4 in hypoxic breast cancer cells by favoring the recruitment of RNA polymerase II, leading to tumor angiogenesis and metastasis. These findings suggest that RAB11B-AS1 may be a target for metastatic breast cancer treatment [34].

CircRNA_103809 is highly expressed in bladder cancer (BC) tissues and cell lines and is associated with poor prognosis in BC patients. CircRNA_103809 knockdown decreases BC cell growth and metastasis. In addition, circRNA_103809 acts as a sponge for mir-516a-5p and promotes FBXL18 expression. Inhibition of circRNA_103809 increases BC cell sensitivity to gemcitabine and may thus help to improve the sensitivity of chemotherapeutic treatments [8]. Using high-throughput sequencing of circular RNAs in renal clear cell carcinoma (RCCC), circRNA-0035483 was found to promote gemcitabine-induced autophagy and enhance RCCC resistance to gemcitabine. Hsa-mir-335 is a regulatory target of circRNA-0035483. CircRNA-0035483 promotes RCCC autophagy and tumor growth by regulating Hsa-mir-335/CCNB1, enhancing RCCC gemcitabine resistance. Inhibiting CircRNA-0035483 can improve gemcitabine sensitivity [8]. Hypoxia-induced lncHILAR acts as a ceRNA for mir-613/206/1-1-3p to elevate renal cancer cell invasion and metastasis, leading to upregulation of Jagged-1 and c-x-cmotif chemokine receptor 4 (CXCR4). Activation of the Jagged-1/Notch/CXCR4 axis can induce RCC cancer invasion and metastasis. Thus, lncHILAR may serve as a potential therapeutic target for renal cell carcinoma [24].

**Figure 2.**
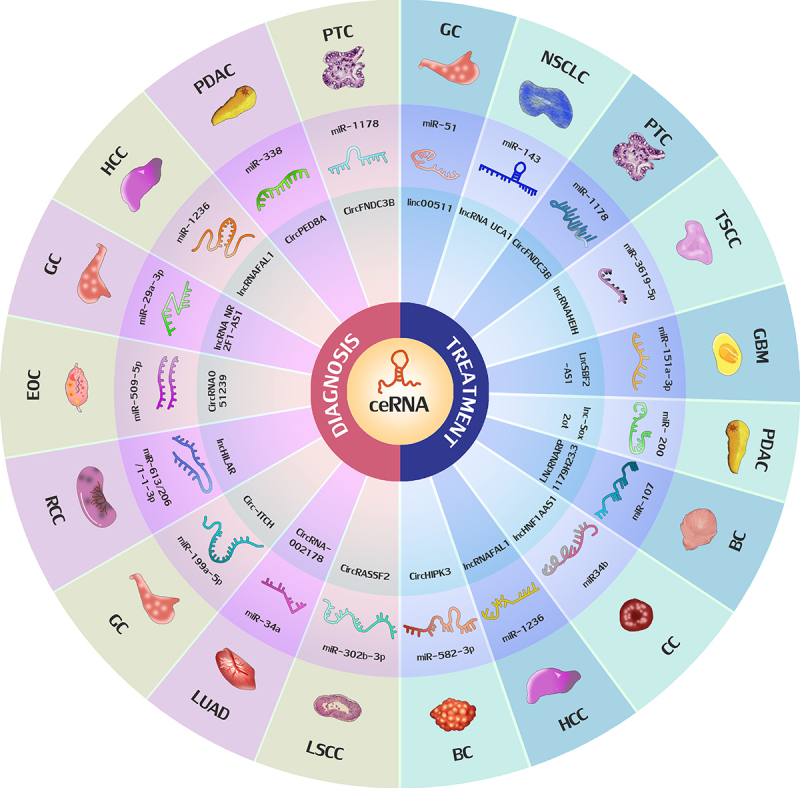
The role of exosomal ceRNA in the diagnosis and treatment of malignant tumors. The red part shows the roles of exosomal ceRNA in the diagnosis of malignant tumors, The blue part shows the roles of exosomal ceRNA in the treatment of malignant tumors. GC, gastric cancer; HCC, hepatocellular carcinoma; GBM: glioblastoma multiforme; PTC: papillary thyroid carcinoma; LSCC, laryngeal squamous cell carcinoma; TSCC: tongue squamous cell carcinoma; GBM: glioblastoma multiforme; PDAC, pancreatic ductal adenocarcinoma;BC:bladder cancer; CC: cervical cancer; RCC: renal cell carcinoma; LUAD: lung adenocarcinoma; EOC: epithelial ovarian cancer; NSCLC, non-small cell lung cancer.

The role of exosomal ceRNA in the diagnosis and treatment of malignant tumors are shown in Figure 2.

## 4. Possible regulatory mechanisms of exosomal ceRNAs in malignant tumors

There is a balance between ceRNA and miRNAs that is closely related to cell species and differentiation and maintained by multiple factors acting against each other [4]. If a ceRNA is expressed differently as a result of genetic or chromosomal mutations, for example, this balance will be disrupted, affecting the expression of miRNA and its target genes, and affecting tumor initiation, progression, invasion, metastasis, and sensitivity to chemotherapeutic agents [9,35].

### 4.1 Cancer promoting mechanisms of ceRNA

Studies show that dysregulated ceRNAs are expressed in many types of cancer, impacting cellular processes, and promoting the occurrence, development, and metastasis of malignant tumors by upregulating proto-oncogenes and pro-oncogenes, downregulating tumor suppressors, promoting the epithelial-mesenchymal transition, and regulating expression of core transcription factors [22]. For example, lncRNAFAL1 is upregulated in hepatocellular carcinoma (HCC) and functions as an oncogene. LncRNAFAL1 promotes cell viability, proliferation, migration, invasion, and epithelial-mesenchymal transition (EMT) by upregulating mir-1236 and inducing expression of AFP and ZEB1 [18]. During cervical cancer development and progression, lncRNA FOXP4-AS1expression is upregulated, and it acts as a mir-136-5p ceRNA to regulate expression of polycomb protein 4 gene (CBX4) and accelerate the development of cervical cancer [36]. Circ-TFF1 was shown to impact breast cancer progression by activating the mir-326/TFF1 axis. While knockdown of circ-TFF1 impaired the migration and EMT processes of breast cancer cells, stimulation enhanced its inhibitory effect on mir-326, reducing mir-326, accelerating tumor cell growth, increasing EMT processes, and promoting breast cancer development and progression [37]. Compared with normal controls, circIFT80 was significantly upregulated in serum exosomes from colorectal cancer (CRC) patients, CRC tissues, and CRC cell lines. circIFT80 was shown to induce CRC invasion and migration through epithelial-mesenchymal transition (EMT), and the growth of colorectal cancer in vivo was inhibited by knockdown of circIFT80 [9].

### 4.2 Tumor suppressor mechanism of ceRNA

CeRNA expression can directly inhibit tumorigenesis by up-regulating tumor suppressor miRNAs [18]. For example, the ceRNA, Mir-1236, acts as a tumor suppressor gene in many cancers, preventing malignant phenotypes by targeting AFP and ZEB1 in liver cancer cells. Similarly, Mir-1236-3p can inhibit migration and invasion by targeting klf8 in lung adenocarcinoma A549 cells [18]. PTEN pseudogene 1 (PTENP1) is a pseudogene of the tumor suppressor PTEN gene, binding to PTEN-specific miRNAs and regulating PTEN expression. In the prostate cancer cell line, DU145, high expression of ptenpi sequesters mr19b and mr-20a, causing PTEN expression levels to rise, inhibiting the downstream PI3K signaling pathway, and suppressing cell growth [4]. Overexpression of ptenpi in renal clear cell carcinoma cell lines that express high levels of mRNA-21 downregulates the effective concentration of mRNA-21, inhibiting tumor cell proliferation and invasion. Growth and metastatic capacity studies have also shown a positive correlation between PTEN and PTENP1 expression in normal human tissues, prostate cancer cell samples, and clinical samples of renal carcinoma, and a negative correlation with corresponding mRNA expression [4].Studies have also found that miR-30a and -b were downregulated in gastric cancer tissue samples and acted as tumor suppressors [38]. In bladder cancer, lncRNA RP11-79H23.3 and the tumor suppressor gene, Pt en, were significantly downregulated in bladder cancer tissues, inhibiting tumorigenesis, angiogenesis, and metastasis, like lncRNA H19. lncRNA RP11-79H23.3 functioned as a ceRNA for mir-107 and inhibited the effect of mir-107 on PTEN. Thus, increased levels of Pt en impedes the ability of PI3K/Akt signaling to inhibit bladder cancer progression [39].

### 4.3 Regulatory mechanisms under hypoxic environment

Tumor angiogenesis is primarily regulated by the hypoxia-inducible factor family, and the imbalance between supplying and consuming oxygen in tumor cells creates a hypoxic environment, especially in patients with advanced tumors. In hypoxic environments, tumors have better growth and are able to secrete more exosomal ceRNAs. Modulation of the tumor microenvironment promotes tumor angiogenesis and metastasis [40]. Studies show that LNC RNAs use multiple mechanisms to stimulate the release of vascular growth factors from tumor cells and this causes endothelial cells to form capillary networks within tumors. Exosomes from the glioma cell line, U87, have higher expression of linc-CCAT2, can deliver linc-CCAT2 to endothelial cells, induce an angiogenic phenotype in vitro and in vivo, and exert anti-apoptotic effects through hypoxia. Linc-CCAT2 upregulates VEGF, TGF- β, and Bcl-2, but downregulates expression of Bax and caspase-3 to inhibit hypoxia-mediated endothelial cell apoptosis. Adenomatous polyposis coli (APC) gene mutations are a key factor during CRC. Wang et al. identified an APC activating incrna-apc1 that impairs tumor growth, metastasis, and angiogenesis. A mechanistic study showed that INCRNA-APC1 is directly associated with rab5bm RNA and upregulating INCRNA-APC1 decreased the stability of its mRNA and production of CRC exosomes. When adenomatous polyposis coli (APC) gene mutations develop into CRC, tumor cells release more exosomes, activate MAPK pathways in endothelial cells, and induce angiogenesis [41]. These findings suggest that exosomal ceRNAs are important elements of the tumor microenvironment as messengers that transmit signals between hypoxic and normoxic renal cell carcinoma cells. Through exosomes, renal carcinoma cells in hypoxic regions may render recipient renal carcinoma cells in normoxic regions more aggressive. Hypoxia can induce lncHILAR expression, which acts as a ceRNA of mir-613/206/1-1-3p to competitively activate the Jagged1/Notch/CXCR4 pathway, thereby promoting renal carcinoma cell invasion, migration in vitro, and metastasis in vivo. Hypoxic renal cell carcinoma cells can also secrete exosomes packaged with lncHILAR. Exosomes secreted from hypoxic renal cell carcinoma cells can be taken up by recipient normoxic RCC cells which acquire exogenous lncHILAR to develop a cell invasive phenotype. Thus, lncHILAR under hypoxia may be an important regulator of renal cell carcinoma invasion and metastasis. A recent study found that hypoxia can promote the release of exosomes in breast cancer cells. In addition, hypoxic cancer cell-derived exosomes may induce angiogenesis by modulating the phenotype of endothelial cells [24].

### 4.4 Regulatory mechanisms of chemosensitivity

Studies have shown that ceRNAs modulate chemosensitivity by direct sponging, repression, and epigenetic mechanisms. For example, through tumor heterogeneity, cancer cells can exhibit primary or inherent chemoresistance, or secondary or acquired chemoresistance through target cell inactivation or alteration, inhibition of cell death, epigenetics, EMT, and other mechanisms [42]. Recent studies show that lncRNA can be enriched in exosomes, which are secreted into body fluids by tumor cells [43]. Extraction analysis of exosomes from 86 gefitinib-treated NSCLC patients showed that lncRNA UCA1 was more highly expressed in extracted serum exosomes, and inhibited the effect of gefitinib by targeting the miR-143 pathway, helping to promote acquired resistance [44]. Zhang et al [45]. Found that knockdown of lncRNA UCA1 inhibited resistance to gefitinib during treatment by targeting STAT3 signaling. Moreover, exosomal lncRNAheih promotes cisplatin resistance in tongue squamous cell carcinoma by targeting mir-3619-5p/HDGF axis. lncHNF1AAS1 was upregulated in the DDP resistant cell line, HeLa/DDP, and silencing lncHNF1AAS1 was shown to inhibit cervical cancer (CC) cell proliferation and promote apoptosis. Lnchnf1aas1 acts as a ceRNA for mir34b, promoting tuft1 expression and downregulating mir34b, inducing CC cell proliferation and drug resistance and inhibiting apoptosis [32].The circRNA, Cdr1as, was downregulated in ovarian cancer cisplatin-resistant patient tissues and cell lines, demonstrating the regulatory mechanism of the mir-1270/SCAI signaling pathway. These results indicated that Cdr1as could improve cisplatin sensitivity of ovarian cancer cells, and can act as a molecular sponge for mir-1270, impairing the inhibitory effect of miRNAs on downstream target genes SCAI. Furthermore, the dual-luciferase reporter system and rip assay validated the direct interaction between Cdr1as, mir-1270, and SCAI. These results suggest that Cdr1as may regulate the sensitivity of ovarian cancer cells to cisplatin and promote ovarian cancer progression [46,47].

## 5. Conclusion

The ceRNA hypothesis has been extensively studied since it was proposed in 2011, particularly its role in regulating tumor progression. Roles and possible mechanisms of exosomal ceRNAs in malignant tumors are shown in Table 1. The regulatory details of how ceRNA is involved in malignant tumor processes require further study, gradually unveiling the post-transcriptional regulatory network of genes in both tumors and normal tissues and allowing for an improved understanding of the occurrence and development of tumors, including how ceRNA is carried by exosomes from the cell of origin. It is expected that ceRNA will be widely used to assist in the early detection and clinical diagnosis of tumors, help predict tumor prognosis and treatment outcomes and develop new antitumor drugs and clinical tumor interventions. Future studies of malignant tumor-derived exosomes ceRNAs will greatly enhance the use of biomarkers and tumor immunotherapy and promote the advancement of translational medicine.

**Table 1.** Roles and possible mechanisms of exosomal ceRNAs in malignant tumors

Cancer types	ceRNA	Expression level	Techniques of regulation		Downstream regulators	Target molecules	Biological function	Predictive use	References
LSCC	CircRASSF2	Up	Sponging	miR-302b-3p		IGF-1 R	Regulation of tumor cell proliferation, invasion, migration	Diagnosis, targeted therapy	15
GBM	LncSBF2-AS1	Up	Sponging	miR-151a-3p		XRCC4	Regulation of tumor chemosensitivity	Targeted therapy	26
TSCC	lncRNAHEIH	Up	Sponging	miR-3619-5p		HDGF	Regulation of tumor chemosensitivity	Targeted therapy	25
PTC	CircFNDC3B	Up	Sponging	miR-1178		TLR4	Regulation of tumor proliferation, migration, invasion, and EMTC	Targeted therapy	16
LUAD	CircRNA- 002178	Up	Sponging	miR-34a		PDL1	Induction of T cell exhaustion	Early diagnosis	17
NSCLC	lncRNA UCA1	Up	Sponging	miR-143		MYO6	Regulation of tumor chemosensitivity	Targeted therapy	28
PDAC	lnc-Sox2ot	Up	Competitively binds	miR- 200		Sox2	Regulation of tumor proliferation, migration, invasion, and EMTC	Targeted therapy	47
GC	linc00511	Up	Sponging	miR-51		EZH2	Regulation of tumor cell proliferation and migration	Potential drug targets	43
GC	Circ-ITCH	Down	Sponging	miR-199a-5p		Klotho	Regulation of tumor cell invasion and migration	Tumor diagnosis	20
HCC	lncRNAFAL1	Up	Competitively binds	miR-1236		ZEB1/AFP	Regulation of tumor chemosensitivity	Targeted therapy	18
RCC	lncHILAR	Up	Sponging	miR-613/206/1-1-3p/Jagged-1/Notch		CXCR4	Regulation of tumor proliferation and invasion	Early diagnosis, Targeted therapy	24
BC	INcRNARP1179H23.3	Down	Sponging	miR-107		PTEND	Regulation of tumorigenesis, angiogenesis and metastasis	Targeted therapy	39
BC	CircHIPK3	Up	Directly binds	miR-124		-	Regulation of tumor cell proliferation and migration	Targeted therapy	42
BC	lncRNA TUSC8	Down	Sponging	miR-190b-5p		MYLIP	Regulation of tumor cell proliferation, invasion, migration	Targeted therapy	40
BC	CircHIPK3	Up	Sponging	miR-582-3p		RNF11	Regulation of tumor chemosensitivity	Targeted therapy	33
BC	lncRNA RAB11B-AS1	Up	Sponging	RNA polymerase II		VEGFA/ANGPTL4	Regulation of tumor angiogenesis and metastasis	Targeted therapy	34
EOC	CircRNA051239	Up	Sponging	miRNA-509-5p		PRSS3	Regulation of tumor cell proliferation, invasion, apoptosis, etc	Targeted therapy	44
OC	CircRNACdr1as	Down	Sponging	miR-1270		-	Regulation of tumor chemosensitivity	Targeted therapy	46
CC	lncHNF1AAS1	Up	Competitively binds	miR34b		TUFT1	Regulation of tumor chemosensitivity	Targeted therapy	32

**Abbreviations**: OC,ovarian cancer.
