# Correction

**DOI:** 10.1080/21655979.2026.2723625

**Published:** 2026-10-01

**Article title**: Role of exosomal competitive endogenous RNA (ceRNA) in diagnosis and treatment of malignant tumors.

**Authors**: Mao, M., Zhang, J., Xiang, Y., Gong, M., Deng, Y., & Ye, D.

**Journal**: *Bioengineered*

**Bibliometrics**: *13*(5), 12156–12168

**DOI**: 10.1080/21655979.2022.2073130

Several in-text citations have been amended to the correct version and one additional citation has been added. The consent for publication has also been amended. Please see below for the list of corrections:

1) In Section 3.1, 5th para:

CircRNA051239 expression was significantly enhanced in ovarian cancer tissues and plasma exosomes. Studies have elucidated the mechanism by which exosomal circular rna051239 promotes tumor progression of EOC, and circRNA051239 provides a tumor marker for epithelial ovarian cancer (EOC) [22].

**Correction**: [22] has been changed to [44].

2) In Section 3.2, 2nd para:

The ceRNA network also plays a role in the regulation of immune responses in papillary thyroid carcinoma (PTC) and several miRNAs, such as mir-355-5p, mir-328-3p, mir-183-3p, and mir- 29b-2-5p, have been found to suppress activity in PTC. Some of these miRNAs, like mir-7-5p, also have specific immune regulatory profiles. Its target mRNAs acted on recombinant human cardiotrophin-like factor 1 (CLCF1) and insulin receptor substrate 2 (IRS2), and these mRNAs also appeared in the mir-7-5p-mediated ptcceRNA network, suggesting that the PTC immune response was regulated by the ceRNA network. The key RNAs in these ceRNA networks may impact immunotherapeutic outcomes and serve as potential biomarkers [22].

**Correction**: [22] has been changed to [48] and the following new reference added to the reference list: 48. Huang CT, Oyang YJ, Huang HC, et al. MicroRNA-mediated networks underlie immune response regulation in papillary thyroid carcinoma. Scientific Reports. 2014;4:6495 [https://doi.org/10.1038/srep06495]

3) In Section 3.2, 6th para:

CircRNA051239 expression was significantly enhanced in ovarian cancer tissues and plasma exosomes. In addition, circRNA051239 is contained in exosomes released by highly metastatic ovarian cancer SKOV3 cells. IP cells promoted proliferation, stimulated migration, and invasion of low metastatic ovarian cancer SKOV3 cells. Studies show that circRNA051239 affects prss3 expression by sponging mir-509-5p. Recent studies have described the mechanism by which circRNA051239 promotes tumor progression of EOC and provides a potential therapeutic target [22].

**Correction**: [22] has been changed to [44].

4) In Section 4.4:

Extraction analysis of exosomes from 86 gefitinib-treated NSCLC patients showed that lncRNA UCA1 was more highly expressed in extracted serum exosomes, and inhibited the effect of gefitinib by targeting the miR-143 pathway, helping to promote acquired resistance [44].

**Correction**: [44] has been changed to [28].

5) In Section 4.4:

Zhang et al [45]. Found that knockdown of lncRNA UCA1 inhibited resistance to gefitinib during treatment by targeting STAT3 signaling.

**Correction**: Zhang et al [45]. Found has been changed to Sun et al [45] found.

6) In Section 4.4:

The circRNA, Cdr1as, was downregulated in ovarian cancer cisplatin-resistant patient tissues and cell lines, demonstrating the regulatory mechanism of the mir-1270/SCAI signaling pathway. These results indicated that Cdr1as could improve cisplatin sensitivity of ovarian cancer cells, and can act as a molecular sponge for mir-1270, impairing the inhibitory effect of miRNAs on downstream target genes SCAI. Furthermore, the dual- luciferase reporter system and rip assay validated the direct interaction between Cdr1as, mir-1270, and SCAI. These results suggest that Cdr1as may regulate the sensitivity of ovarian cancer cells to cisplatin and promote ovarian cancer progression [46,47].

**Correction**: [46,47] has been changed to [46].

7) Consent for publication declaration:

All authors have agreed on the contents of the manuscript.

**Correction**: the whole sentence has been changed to Not applicable.

